# Matrix-Bound PAI-1 Supports Cell Blebbing via RhoA/ROCK1 Signaling

**DOI:** 10.1371/journal.pone.0032204

**Published:** 2012-02-21

**Authors:** Amandine Cartier-Michaud, Michel Malo, Cécile Charrière-Bertrand, Gilles Gadea, Christelle Anguille, Ajitha Supiramaniam, Annick Lesne, Franck Delaplace, Guillaume Hutzler, Pierre Roux, Daniel A. Lawrence, Georgia Barlovatz-Meimon

**Affiliations:** 1 IBISC EA 4526, Evry Val d'Essonne University, Evry, France; 2 University Paris-Est Créteil, Créteil, France; 3 CRBM UMR 5237 CNRS, Montpellier University, Montpellier, France; 4 Institut des Hautes Etudes Scientifiques, Bures-sur-Yvette, France; 5 LPTMC UMR 7600 CNRS, Paris, France; 6 Department of Internal Medicine, University of Michigan School of Medicine, Ann Arbor, Michigan, United States of America; Institut national de la santé et de la recherche médicale - Institut Cochin, France

## Abstract

The microenvironment of a tumor can influence both the morphology and the behavior of cancer cells which, in turn, can rapidly adapt to environmental changes. Increasing evidence points to the involvement of amoeboid cell migration and thus of cell blebbing in the metastatic process; however, the cues that promote amoeboid cell behavior in physiological and pathological conditions have not yet been clearly identified. Plasminogen Activator Inhibitor type-1 (PAI-1) is found in high amount in the microenvironment of aggressive tumors and is considered as an independent marker of bad prognosis. Here we show by immunoblotting, activity assay and immunofluorescence that, in SW620 human colorectal cancer cells, matrix-associated PAI-1 plays a role in the cell behavior needed for amoeboid migration by maintaining cell blebbing, localizing PDK1 and ROCK1 at the cell membrane and maintaining the RhoA/ROCK1/MLC-P pathway activation. The results obtained by modeling PAI-1 deposition around tumors indicate that matrix-bound PAI-1 is heterogeneously distributed at the tumor periphery and that, at certain spots, the elevated concentrations of matrix-bound PAI-1 needed for cancer cells to undergo the mesenchymal-amoeboid transition can be observed. Matrix-bound PAI-1, as a matricellular protein, could thus represent one of the physiopathological requirements to support metastatic formation.

## Introduction

The amoeboid and mesenchymal modes of migration are both used by cancer cells for moving in their environment and invading the surrounding tissues. Inhibition or up-regulation of specific molecular pathways can determine the choice of migration mode and can also lead to switch to the other type of cell movement, a phenomenon known as mesenchymal-amoeboid transition (MAT) or amoeboid-mesenchymal transition (AMT) [1], [2], [3], [4]. Amoeboid migration is characterized by the presence of round cells and membrane blebbing [5] and requires RhoA and its main effector Rho-associated Coil-containing Protein Kinase 1 (ROCK1), which regulates the phosphorylation of Myosin Light Chain (MLC) and Acto-Myosin contractility during the bleb life cycle [5], [6], [7]. Moreover, 3-Phosphoinositide-Dependent-Protein-Kinase 1 (PDK1), an important regulator of cortical MLC phosphorylation, indirectly activates ROCK1 at the plasma membrane and thereby promotes amoeboid cell motility [8]. On the other hand and differently from the mesenchymal mode, amoeboid migration does not require pericellular proteolysis [3]. However, the cues that promote the amoeboid behavior in physiological and pathological conditions have not been clearly identified yet [9].

The cell microenvironment can influence both the morphology and behavior of cancer cells (reviewed recently by Mantovani [10]). Plasminogen Activator Inhibitor type-1 (PAI-1) is found in high amount in the microenvironment of aggressive tumors and is considered as a marker of bad prognosis [11], [12]. PAI-1 is part of the Plasminogen Activator (PA) system that includes also urokinase Plasminogen Activator (uPA) and its receptor (uPAR). In addition to catalyzing the degradation of the extracellular matrix and modulating cell adhesion [13], [14], various components of the PA system also influence cell migration [15], [16], [17]. Binding of PAI-1 to Vitronectin (VN) stabilizes PAI-1 in its active conformation. Upon binding to uPAR, PAI-1 decreases its affinity for VN in the matrix and simultaneously increases the affinity for endocytic receptors, such as the low-density Lipoprotein Receptor-related Protein (LRP) [18], [19], [20]. It has been suggested that the urokinase-dependent PA system modulates cell migration through the Ras/ERK pathway and the Rho/ROCK signaling cascade [21], [22]. Numerous studies have shown that uPAR signals through various pathways (Ras-Mitogen-Activated Protein Kinase (MAPK) pathway, Tyrosine kinases, Focal Adhesion Kinase (FAK), Src and Rac GTPase) [23], [24], [25], [26] and a recent review has stressed the role of uPAR, in association with Integrins or Vitronectin, in regulating cell signaling [27]. Although matrix-bound PAI-1 is recognized as a molecule participating in the regulation of the rapid attachment/detachment of cells required for migration [14], [15], [16], [17], [18], [19], no specific signaling linked to this PAI-1 conformation has yet, to our knowledge, been described.

In this study, we focused on the role of immobilized, active PAI-1 in supporting blebbing of SW620 colorectal cancer cells and investigated the signaling cascades involved in PAI-1 promotion of cell blebbing, a typical feature of amoeboid movement. We show that SW620 cells seeded on plates coated with immobilized, active PAI-1 are characterized by more frequent blebbing, colocalization of PDK1 and ROCK1 at the cell membrane and long lasting activation of the RhoA/ROCK1 pathway in comparison to cells seeded on collagen. Moreover, in SW620 cells seeded on immobilized, active PAI-1 we observed membrane depositions. Finally, modeling of the PAI-1 “cycle” (its expression/secretion as a soluble protein, deposition in the matrix, activity or latency, interactions with VN, “consumption” as part of a complex with uPAR and uPA and subsequent internalization) indicates that secreted PAI-1 by tumor cells is not homogeneously distributed at the tumor periphery, but rather localized in high-concentration deposits particularly in tumors characterized by the presence of invaginations [28]. In this study we suggest that the most aggressive cancer cells could “use” these high-concentration spots of matrix-bound PAI-1 to undergo mesenchymal-amoeboid transition and escape using the amoeboid mode of migration; therefore PAI-1 could be part of a new physio-pathological mechanism supporting metastatic escape.

## Results and Discussion

### Immobilized, active PAI-1 supports cell blebbing

**Figure 1:**
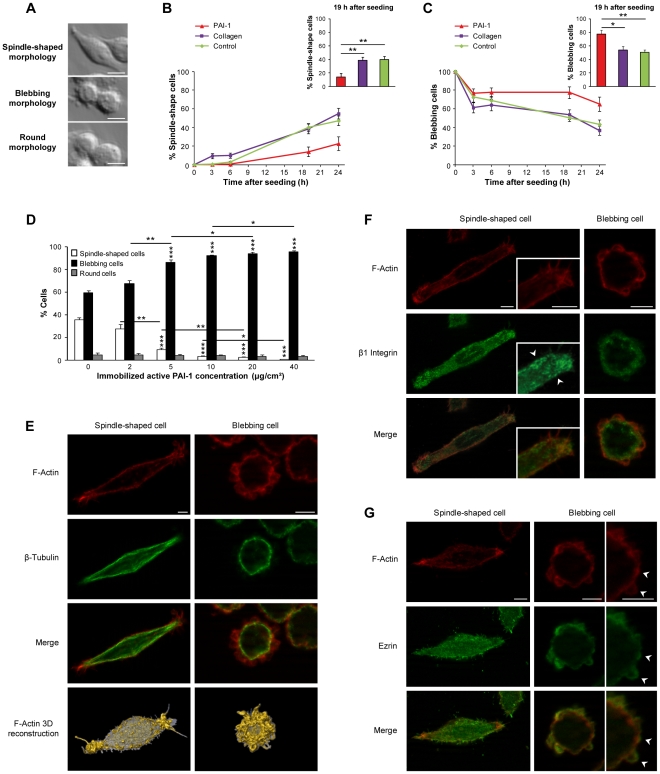
Immobilized, active PAI-1 supports cell blebbing. (**A**) When SW620 human colon cancer cells are cultured in the presence of serum they can be classified in three main morphological types: spindle-shaped, blebbing and round. Bar, 10 µm. The percentage of spindle-shaped (**B**) and blebbing cells (**C**) is influenced by the length of time SW620 cells have been in contact with immobilized, active PAI-1 14-1b, collagen or the matrix secreted by control cells (seeded on non-coated plates). Data are the mean ± s.e.m. of eight independent experiments; *: *P*<0.05, ** *P*<0.01. (**D**) Percentage of spindle-shaped, blebbing and round SW620 cells in plates coated with increasing concentrations of PAI-1 14-1b. Data are the mean ± s.e.m. of four independent experiments; *: *P*<0.05, **: *P*<0.01, ***: *P*<0.001. Analysis by immunostaining and 3D reconstruction of the expression of (**E**) F-Actin and β-Tubulin, (**F**) F-Actin and β1 Integrin (arrowheads point to β1 Integrin clusters), and (**G**) F-Actin and Ezrin (arrowheads point to colocalization in blebs) in blebbing SW620 cell seeded on PAI-1 14-1b and spindle-shaped SW620 cell seeded on collagen at the 19 h time-point after seeding. Bar, 5 µm.

To specifically assess the role of matrix-bound PAI-1 in supporting membrane blebbing of cancer cells, we cultured SW620 human colorectal cancer cells in plates coated with PAI-1 14-1b, a mutant PAI-1 which is active for 500 hours. When cultured on non-coated plates and medium supplemented with serum (multi-molecular environment), SW620 cells could be classified in three main morphological categories: spindle-shaped, blebbing and round cells (Fig. 1A). The proportion of spindle-shaped and blebbing cells was roughly the same (40%). Conversely, when SW620 cells were seeded in plates coated with PAI-1 14-1b or collagen in the absence of serum (mono-molecular environment), their distribution in the three morphological categories differed according to the type of environment. Specifically, the proportion of blebbing cells was always higher in cultures seeded on PAI-1 14-1b than on collagen (Fig. 1B and C, Fig. S1), particularly at the 19 h time-point. This was not due to differences in cell adhesion because the same number of adherent cells was found in both conditions (Fig. S2A). In addition, the number of blebbing cells was proportional to the concentration of PAI-1 14-1b used to coat the plates (from 2 to 40 µg/cm^2^) with saturation of the effect at 20 µg/cm^2^ (Fig. 1D). This concentration was thus used for all the subsequent experiments. Bleb formation/retraction appeared to be a quick process (Video S1), similarly to what has been previously described by others [29], [30]. Experimentally, whatever the microenvironment, blebbing cells showed no β-Tubulin re-organization, but characteristic F-Actin rings in blebs (Fig. 1E). Moreover, clusters of β1 Integrin associated with the F-Actin cytoskeleton were observed at the membrane of spindle-shaped cells, whereas they were absent in blebbing cells (Fig. 1F). Finally, in some blebbing cells, Ezrin expression was restricted to blebs and colocalized with F-Actin [5], [30] (Fig. 1G). This is in coherence with Ezrin role in bleb dynamics [30], i.e. stabilizing newly formed F-Actin-transmembrane protein interactions. In conclusion, the blebbing cells experimentally observed in 2D cultures in the presence of immobilized, active PAI-1 have the main characteristics described by others before [3].

These results indicate that PAI-1 is the first matrix protein supporting cell blebbing in 2D cultures and show that when studying the behavior of cancer cells it is crucial to take into consideration not only the choice of 2D or 3D culture systems [9], but also the nature, conformation and concentration of the molecules present in the environment.

### Immobilized, active PAI-1 maintains the RhoA/ROCK pathway activation

**Figure 2:**
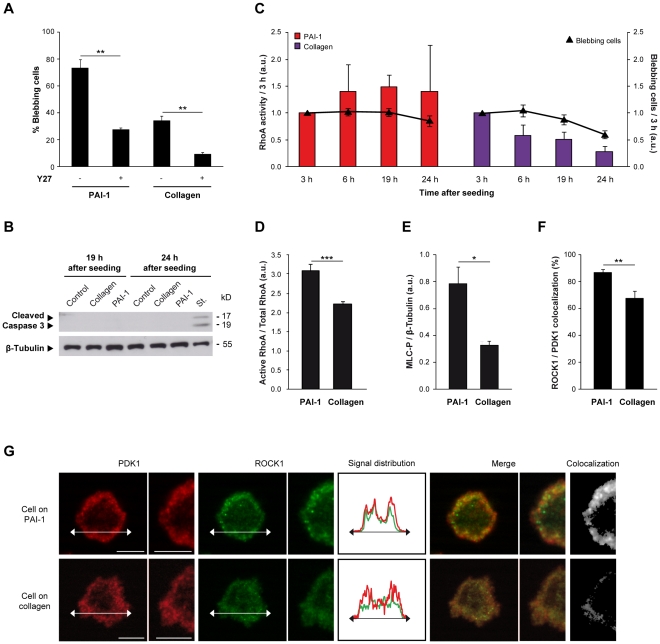
Immobilized, active PAI-1 maintains the RhoA/ROCK pathway activation. (**A**) Percentage of blebbing SW620 cells seeded on PAI-1 14-1b or collagen in the presence of the ROCK inhibitor Y27632 (Y27) (10 µM) at 19 h time-point after seeding. Data are the mean ± s.e.m. of three independent experiments; **: *P*<0.01. (**B**) Detection of cleaved Caspase 3 by immunoblotting in SW620 cells seeded on non-coated plates (control), PAI-1 14-1b or collagen 19 h and 24 h after seeding. SW620 cells treated with 1 µM staurosporin (St.) for 24 h were used as positive control. β-Tubulin served as loading control. (**C**) RhoA activity and proportion of blebbing cells in SW620 cells seeded on PAI-1 14-1b or collagen at different time-points after seeding. Values were normalized to the effects at 3 h for each condition. Data are the mean ± s.e.m. of four independent experiments. (**D**) Quantification following immunoblotting of active RhoA relative to total RhoA in SW620 cells seeded on PAI-1 14-1b or collagen for 19 h. Data are the mean ± s.e.m. of three independent experiments; ***: *P*<0.001. (**E**) Quantification following immunoblotting of phosphorylated MLC (MLC-P) relative to β-Tubulin (loading control) expression in SW620 cells seeded on PAI-1 14-1b or collagen for 19 h. Data are the mean ± s.e.m. of three independent experiments; *: *P*<0.05. (**F**) Percentage of ROCK1 colocalization with PDK1 relative to total ROCK1 expression in blebbing SW620 cells seeded on PAI-1 14-1b or collagen for 30 min. Data are the mean ± s.e.m. of the analysis of ten blebbing cells in each microenvironment; **: *P*<0.01. (**G**) Expression of PDK1 and ROCK1 in blebbing SW620 cells seeded on PAI-1 14-1b or collagen for 30 min. The colocalization of PDK1 and ROCK1 is shown and their signal distributions are compared. Bar, 5 µm.

As blebbing can occur in a ROCK1-dependent manner [31], we then assessed the effects of Y27632, a ROCK inhibitor, on the morphology of SW620 cells seeded either on PAI-1 14-1b- or on collagen-coated plates. As expected, addition of Y27632 caused a dramatic decrease in the number of blebbing cells (Fig. 2A) and an increase of spindle-shaped cells (data not shown), indicating that the observed blebbing process is ROCK-dependent. Then we examined the two main pathways leading to ROCK activation: Caspase 3 and RhoA [31]. In our experimental conditions, ROCK activation was Caspase 3-independent, with no apoptosis as attested by the absence of cleaved Caspase 3 (Fig. 2B), of DNA cleavage and by the normal growth curves of cells seeded on PAI-1 14-1b or collagen (Fig. S2B). Conversely, RhoA was induced in blebbing cells 10 min after seeding in both experimental conditions (data not shown). However, while in blebbing cells seeded on PAI-1 14-1b, RhoA activation increased with time till the 19 h time-point (Fig. 2C), in blebbing cells seeded on collagen, RhoA activation decreased rapidly after the 3 h time-point concomitantly with the number of blebbing cells (Fig. 2C). Accordingly, at the 19 h time-point, RhoA activity was higher in SW620 cells seeded on PAI-1 14-1b than in those seeded on collagen (Fig. 2D). Similarly, the amount of phosphorylated MLC (MLC-P) at the 19 h time-point was also higher in cells seeded on PAI-1 14-1b than in cells seeded on collagen (Fig. 2E). Finally, quantification by using the Axiovision 4.5 “Colocalization” module (Zeiss) indicated that ROCK1 and PDK1 were more frequently colocalized at the membrane in SW620 cells seeded on PAI-1 14-1b than in cells seeded on collagen. This phenomenon was more evident at the 30 min (Fig. 2F) than at the 19 h time-point (Fig. S3A). Specifically, as shown by the illustrative example in Figures 2G and S3A, at any time-point, ROCK1 was preferentially localized near the cell membrane in SW620 cells seeded on PAI-1 14-1b, while it was homogeneously distributed in the whole cell in cells seeded on collagen. Conversely, PDK1 was always localized at the cell membrane whatever the environment and the time-point (Fig. 2G, Fig. S3A). These observations are in agreement with the need to recruit PDK1 and ROCK1 to the cell membrane to allow cell blebbing, as suggested by Pinner and Sahai [8]. These results indicate that immobilized, active PAI-1 supports cell blebbing through activation of the RhoA/ROCK1/MLC-P pathway and colocalization of PDK1 and ROCK1 at the cell membrane. This finding could help understanding how PAI-1 contributes to cancer development and progression.

### The Plasminogen Activator system influences cell blebbing

**Figure 3:**
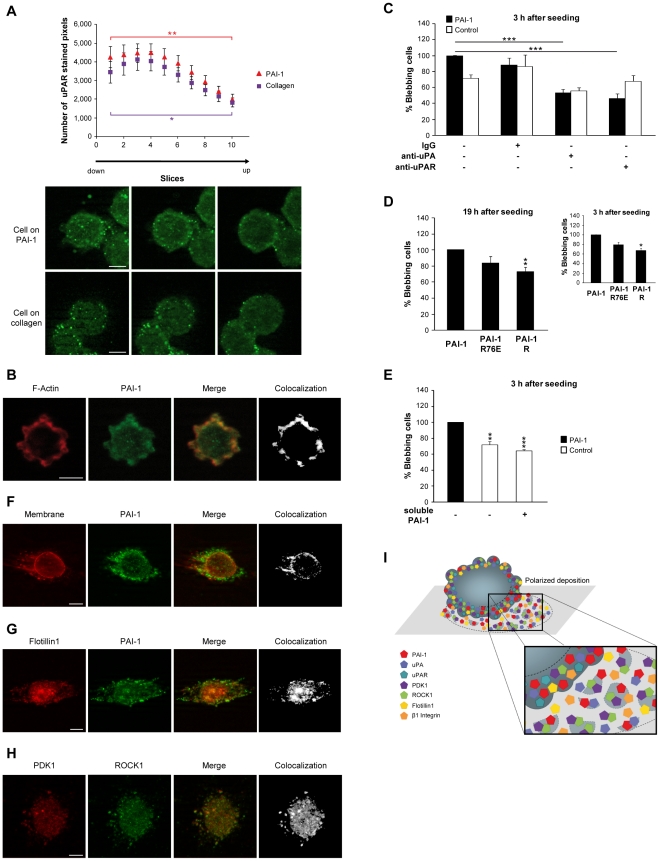
The Plasminogen Activator system influences cell blebbing. (**A**) Number of stained pixels of the uPAR immunostaining z-profile in SW620 cells seeded on PAI-1 14-1b or collagen for 19 h. Data are the mean ± s.e.m. of the analysis of thirty blebbing cells; *: *P*<0.05, **: *P*<0.01. (**B**) Expression of F-Actin and PAI-1 in blebbing SW620 cells seeded on PAI-1 14-1b for 19 h. (**C**) Percentage of blebbing SW620 cells (3 h after seeding) seeded on plates coated with PAI-1 14-1b or non-coated plates (Control) and in which the medium was supplemented with IgG, anti-uPA or anti-uPAR antibodies. Data are the mean ± s.e.m. of three independent experiments; ***: *P*<0.001. (**D**) Percentage of blebbing SW620 cells seeded on PAI-1 14-1b, PAI-1 R76E or PAI-1 R at the 3 h and 19 h time-points. Data are the mean ± s.e.m. of three independent experiments; *: *P*<0.05, **: *P*<0.01. (**E**) Percentage of blebbing SW620 cells (3 h after seeding) seeded on plates coated with PAI-1 14-1b or non-coated plates (Control) and in which the medium was supplemented with soluble PAI-1. Data are the mean ± s.e.m. of three independent experiments; **: *P*<0.01, ***: *P*<0.001. (**F**) Localization of PAI-1 at the membrane (stained with wheat germ agglutinin) in SW620 cells seeded on non-coated plates for 30 min. (**G**) Flotillin1 and PAI-1 expression in SW620 cells seeded on non-coated plates for 30 min. (**H**) PDK1 and ROCK1 expression in SW620 cells seeded on PAI-1 14-1b for 30 min. (**I**) Schematic representation of deposits, which include PAI-1, uPA, PDK1, ROCK1, Flotillin1 and β1 Integrin, of blebbing cells in a 2D microenvironment. Bar, 5 µm.

The PA system has been involved in cancer progression through its proteolytic activity via uPA, by regulating cell adhesion via PAI-1 [13], through its involvement in mechanical transmission [14], [32] and finally through its role in cell migration [15], [17], [33] (Fig. S4). SW620 cells seeded on PAI-1 14-1b or collagen expressed uPA and uPAR, but the distribution of uPAR in blebbing cells was not homogenous. Indeed, uPAR expression was higher in the basal part of the cells and this could be of importance for cells on matrix-bound PAI-1 (Fig. 3A). Moreover, in blebbing cells, uPAR colocalized at the cell membrane with uPA (Fig. S3B) and PAI-1 (Fig. S3C), which was strongly expressed in blebs (Fig. 3B), suggesting that [uPAR∶uPA∶PAI-1] complexes might be more easily formed at the membrane of cells in such conditions.

We then tried to understand to which extent the formation of [uPAR∶uPA∶PAI-1] complexes is needed for maintaining cell blebbing. To this aim, we first tried to impair the formation or internalization of the [uPAR∶uPA∶PAI-1] complex by using antibodies against uPA and uPAR. Addition of anti-uPA or anti-uPAR antibodies to the culture medium led to a significant decrease in the number of blebbing cells in cultures seeded on PAI-1 14-1b (Fig. 3C). We then grew SW620 cells in plates coated with PAI-1 R, a dominant negative PAI-1 mutant that binds transiently and weakly to uPA [34]. Also in this case the number of blebbing cells decreased (Fig. 3D). However, addition of soluble PAI-1 14-1b had no effect on the blebbing of cells seeded on non-coated plates (Fig. 3E), thus ruling out a positive influence of soluble PAI-1 on the blebbing process and stressing the specific role of matrix-bound PAI-1 in the same process. Taken together, these data suggest that the optimal situation for cancer cell blebbing is the presence of uPAR together with active uPA and immobilized, active PAI-1. LRP does not seem to be directly required for cell blebbing because seeding cells on plates coated with PAI-1 R76E, a PAI-1 mutant that cannot bind to LRP, only slightly modified the number of blebbing cells (Fig. 3D). However, we cannot exclude the participation of other internalization receptors, such as Endo 180 or GP 330, in uPAR recycling [35], [36], [37]. Moreover, the localization of uPAR at the cell membrane of blebbing cells suggests that uPAR could participate in the process of polarization involved in cell amoeboid migration. Indeed, it has been stressed [27] that the multiple functions and interactions of uPAR and uPA must be taken into account when trying to understand the process of cancer cell migration, especially the finely tuned crosstalk between cancer and stromal cells (in terms of changes in the microenvironment).

Finally, at early time-points after seeding on non-coated plates, little spots that were strongly positive for PAI-1 expression were observed around the membrane of blebbing cells on immobilized, active PAI-1 and non-coated plates in about 30% of the entire cell population, but not on collagen coated plates (Fig. 3F, Video S2). This suggests that membrane depositions are formed at the membrane of blebbing cells, as previously described in conventionally migrating cells [38], [39], [40] and in blebbing cells [41]. Membrane depositions can influence processes as diverse as cell polarity, differentiation, migration, chemotherapy resistance, immuno-regulation, inflammation, coagulation, angiogenesis and cancer metastasis [42] and to act as mediators of intercellular communication in cancer [42]. Colorectal cancer cells, among which also SW620 cells, can release exosome-like membrane depositions [43], [44]. Proteomic analysis of these structures in SW620 cells showed the presence of PAI-1 and uPA, but no uPAR, PDK1 or ROCK1 (Lim JWE et al., 2010, http://www.ludwig.edu.au/archive/JustinLimSupplemental/index.htm). Similarly, in our experimental conditions, membrane depositions contained PAI-1 (Fig. 3F and G, Fig. S3D, Video S2 and Video S3) and uPA (Fig. S3F), but not uPAR. However, both PDK1 and ROCK1 were found in these structures (Fig. 3H, Video S4), as well as Flotillin1 (Fig. 3G, Fig. S3E) and β1 Integrin (Fig. S3D and E) that have been previously detected in exosomes and ectosomes. Moreover, PAI-1 strongly colocalized with the cell membrane (labeled with fluorescent wheat germ agglutinin), decorated membrane deposits, and partially colocalized with Flotillin1 and β1 Integrin (Fig. 3F and G, Fig. S3D).

Such membrane depositions were never observed in cells seeded on collagen (data not shown), suggesting that immobilized, active PAI-1 could act as an indirect promoter of accumulation at the cell membrane of various molecules and vesicle/membrane deposition by supporting blebbing, which in turn also favors accumulation at the cell membrane and vesicle/membrane deposition. Matrix-bound PAI-1 thus is, to our knowledge, the first matricellular protein [45] to influence the formation of such membrane depositions. Moreover, PAI-1 in membrane deposits was also detected with an antibody directed against active PAI-1 (data not shown), thus suggesting a unique role for blebbing in the microenvironmental modification process. Furthermore, membrane deposits formation and accumulation appeared to be polarized (Fig. 3F, G and H), as schematically summarized in Figure 3I. Each cell might thus participate in the modification of its immediate environment, including the presence of spots of active PAI-1 in the matrix, defining qualitatively and topologically a new microenvironment for itself but also for other cells, as stressed in previous studies [46].

**Figure 4:**
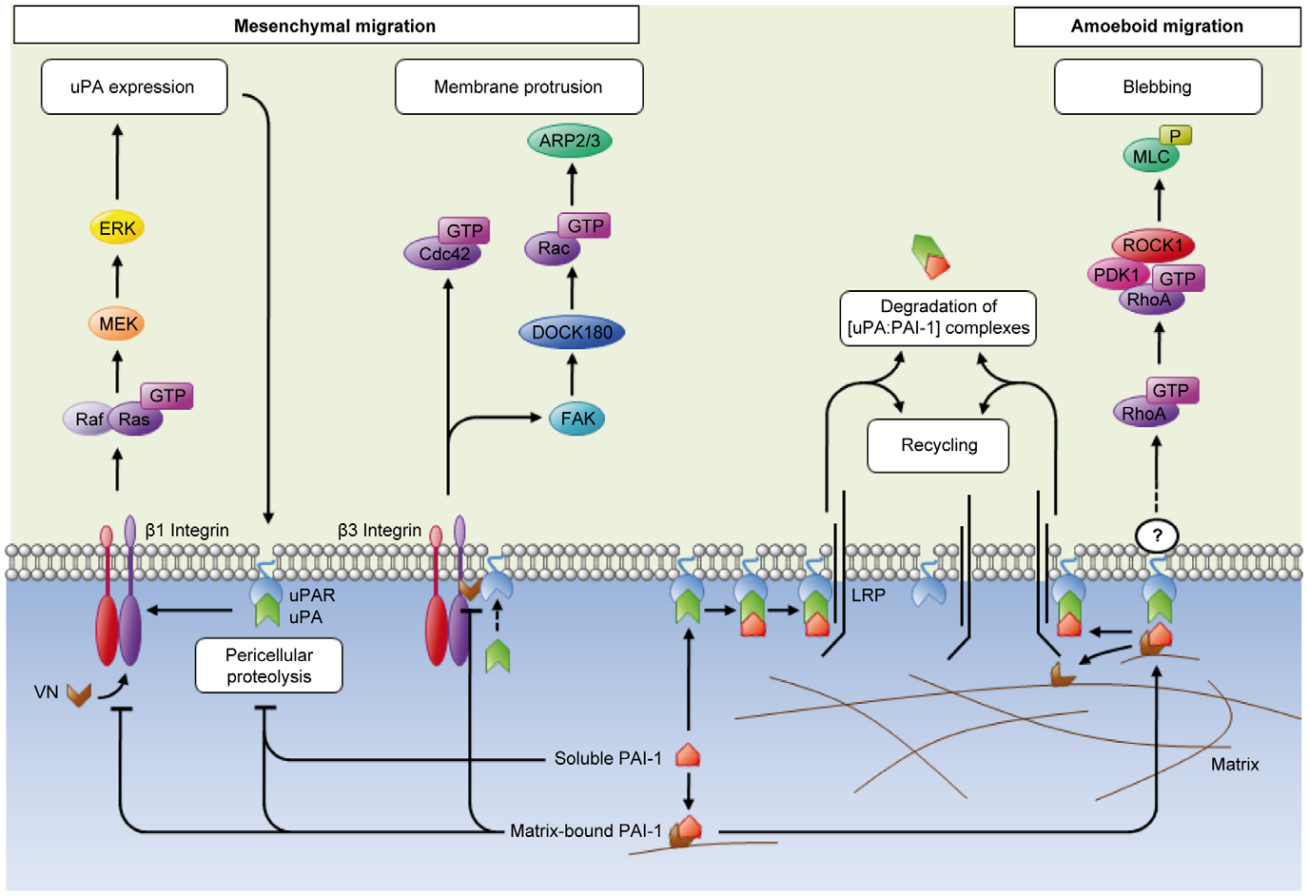
Involvement of the Plasminogen Activator system in the signaling to regulate the transition between mesenchymal and amoeboid migration. uPAR can interact with different types of Integrins and participates in the activation of the MEK/ERK and Rac pathways, both leading to mesenchymal migration. Active, matrix-bound PAI-1 blocks the association of Integrin and uPAR with the extracellular matrix and subsequently supports cell blebbing by activating the RhoA pathway. If only matrix-bound PAI-1 is available, as in our experimental model, the RhoA pathway is activated either through biomechanical transmission (implicating an unknown co-actor) or transduction (implicating a transmembrane receptor; altogether these events might lead to amoeboid migration.

Altogether our work suggests that, in SW620 cells, formation of [uPAR∶uPA∶PAI-1] complexes, particularly with matrix-bound PAI-1, is pivotal in supporting cell blebbing. The higher uPAR expression in the basal part of cells seeded on PAI-1 14-1b (Fig. 3A) suggests that such complexes are more likely to be formed in the adherent part of the cell. This is well in line with previous studies showing that VN expression is localized between the cell membrane and the extracellular matrix [47]. As [uPAR∶uPA∶PAI-1] complex formation is needed to activate the Rho pathway and to support cell blebbing and since uPAR is not a transmembrane receptor, we think that this effect might not occur through direct signal transduction (which requires a transmembrane receptor), but rather through biomechanical signal transmission (thus implicating an unknown co-actor), as previously mentioned [32] (Fig. 4). The role of matrix-bound PAI-1 in blebbing could therefore be to promote many new and weak interactions between matrix-bound [uPAR∶uPA∶PAI-1] complexes and cofactors at the cell membrane, that might lead to amoeboid cell behavior.

### Modeling PAI-1 deposition and simulation of its possible effects on amoeboid cell behavior

**Figure 5:**
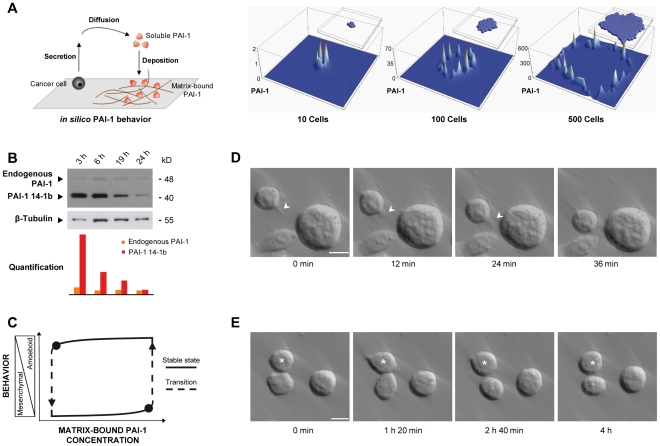
Modeling PAI-1 deposition and simulation of its possible effects on amoeboid cell behavior. (**A**) Cellular-automata simulation of PAI-1 secretion, diffusion and deposition (vertical axis) in tumors of different cell size (10, 100 and 500 cells). (**B**) Detection of endogenous PAI-1 and recombinant PAI-1 14-1b by immunoblotting in SW620 cells seeded on PAI-1 14-1b at different time-points. Signal intensities were normalized to the expression of β-Tubulin. (**C**) Mathematical modeling of the possible effects of changes in the concentration of matrix-bound PAI-1 in the hysteretic cycle between amoeboid and mesenchymal behavior. (**D**) Amoeboid pseudopodal migration of a blebbing SW620 cell seeded on PAI-1 14-1b (17 h after seeding). Arrowhead indicates a pseudopod. Speed was evaluated at 0.4 µm/min (see also Video S6). (**E**) Transition between blebbing and spindle-shaped morphology, and back, in blebbing morphology in a SW620 cell (★) seeded on PAI-1 14-1b (7 h after seeding) (see also Video S7). Bar, 10 µm.

In order to durably maintain membrane blebbing, PAI-1 has to be present in the matrix at very high concentrations. To further investigate the scenario suggested by our experimental observations, we first carried out an *in silico* simulation aiming at modeling PAI-1 deposition around a growing tumor (Fig. 5A) by implementing a cellular automata simulation of PAI-1 secretion by growing tumor cells. This model showed that a minimal mechanism, i.e. the random growth of tumor cells together with the diffusion/deposition of secreted PAI-1 (or of any secreted protein) on the available matrix, is enough to account for the highly heterogeneous distribution (spots) of matrix-bound PAI-1 at the tumor periphery. Then we used a more refined simulation to describe how the behavior of cancer cells is modified by the presence of increasing/decreasing concentration of immobilized, active PAI-1. This is a more “realistic” agent-based simulation of tumor growth, involving more cell states and different molecular species, although still simpler than the biological reality and restricted to what we think to be the main actors (Fig. S5, Video S5). It reproduces both the observed structure of a tumor and its surroundings as well as the heterogeneous distribution of matrix-bound PAI-1 around the tumor [12]. A given cell at the periphery of the tumor can “meet” a spot of high concentration of matrix-bound PAI-1 and “use” it for maintaining blebbing. Overall, both simulation studies support a mechanism whereby all tumor cells produce and deposit enough PAI-1 to maintain the blebbing of a very small fraction of them. This behavior is favored by the local morphology and tumor growth history, thus explaining the persistence of a microenvironment locally favorable to blebbing.

This feature of the tumor microenvironment can be pivotal in modulating (or influencing) the behavior of a single cell following variation of the concentration of available matrix-bound PAI-1. In Figure 5C, we describe the dynamical transitions between the mesenchymal and amoeboid state of a single cell depending on the microenvironment. This two-state diagram is supported by our experimental data, showing that, in the presence of high concentrations of matrix-bound PAI-1, more cells are in the amoeboid state (Fig. 1D and Fig. S1), a situation associated with the activation of the RhoA/ROCK1/MLC-P signaling pathway. We thus hypothesized that, when the extracellular matrix of a tumor cell is progressively enriched in PAI-1 (x-axis), the cell mesenchymal state maintained by adhesion via Integrins (y-axis) is perturbed. Indeed, as observed by others, PAI-1 is an efficient perturbator of Integrin-dependent adhesion [19], [48]. Specifically, high concentrations of PAI-1 disrupt the first equilibrium state and drive the cell towards another equilibrium, i.e. the amoeboid state where adhesion becomes PAI-1-dependent. If all the required conditions are met, the cell starts to move on matrix-bound PAI-1, “consumes” it by internalizing it together with [uPA∶uPAR] complexes and leaves the high-concentration PAI-1 spot [28]. Experimentally, cells seeded on immobilized, active PAI-1 progressively consume it as indicated by the time-dependent decrease of PAI-1 14-1b concentration (and effect), while their production of endogenous PAI-1 is not modified (Fig. 5B). As a consequence, cell spreading is again favored by reengagement of Integrin-dependent adhesion (in our conditions, after the 19 h time-point, Fig. 1B), until the cell reaches a new equilibrium, i.e. the mesenchymal state. These MAT and AMT transitions are mathematically modeled as a switch between two dynamically stable states associated with amoeboid and mesenchymal behavior (Fig. 5C). The changes at different matrix-bound PAI-1 concentrations correspond to the hysteretic cycle predicted by our model. Our integrated scenario for metastatic escape is based on a succession of “hiving cycles” in which the microenvironment is controlled internally by the tumor growth and secretions, switching cells (when the matrix-bound PAI-1 concentration is high enough) from the mesenchymal state to the amoeboid state. This allows the cell to escape from the primary tumor and, possibly, to generate a secondary tumor, when switching back to the mesenchymal state (at low concentrations of matrix-bound PAI-1). Although migration of blebbing cells is an individual and rare phenomenon, as illustrated by the presence of a single cell migrating towards other cells in a PAI-1-coated well seeded with SW620 cancer cells (Fig. 5D, Video S6), cycling between amoeboid and mesenchymal behavior could explain early cancer cell escape, in which phases of proliferation and migration (leading to metastasis) can take place [28].

Based on this model we might also hypothesize that the most aggressive cancer cells could “use” the spots of high concentration of matrix-bound PAI-1 to undergo mesenchymal-amoeboid transition, following a physiological hysteresis [28], to deposit membrane depositions and to escape using the amoeboid mode of migration. VN is probably deposited in a similar fashion (i.e., in the extracellular matrix around some cell clusters) [49], thus offering multiple binding sites to PAI-1, which can be stabilized in its active conformation.

It is now recognized that the blebbing and mesenchymal states both involve rapid changes that are viewed as a response to specific cues from the microenvironment [1]. Experimentally, a blebbing cell can spread and then return to the blebbing state (Fig. 5E, Video S7), or stay spread (Video S8).

Our results highlight that amoeboid cell migration is supported by matrix-bound PAI-1 in a 2D environment. It is predominantly “single cell, blebby, covering local short distances”, and, as such, consistent with the way cancer metastases reach distant sites [9], [50]. However, the PAI-1 effect on cell blebbing is observed only when PAI-1 is present at high concentration, in the active conformation and immobilized; moreover, PAI-1 binding to LRP or its internalization does not seems to be indispensable based on our observations. The availability of uPA is required for binding to matrix-bound PAI-1, which is its immediate, localized and preferred inhibitor, rather than for its proteolytic activity [51]. The formation of [uPAR∶uPA∶PAI-1] complexes leads to weak adhesion [32], but rapid turnover [28], especially of uPAR which seems to be localized in blebs, like ROCK1 and PDK1, leading to the local persistence of blebbing [8].

All together our results show that blebbing cells in contact with high concentrations of matrix-bound PAI-1 extend the duration of blebbing and display the main requirements for amoeboid behavior. A tumor environment rich in matrix-bound PAI-1 might thus provide interconnected inputs that modulate cell adhesion and cytoskeletal organization, impacting on tumor cell shape, cell polarization and finally, mode of migration. PAI-1 is the first matrix-bound protein described as maintaining membrane blebbing of cancer cells supporting amoeboid behavior, and thereby most possibly favoring metastatic escape.

## Materials and Methods

### Cell line and microenvironments

The SW620 human colorectal cancer cell line (from ATCC) was maintained in Leibovitz L-15 medium (Invitrogen) supplemented with 10% fetal calf serum (FCS) (Sigma-Aldrich), 100 U/ml penicillin and 100 µg/mL streptomycin (Invitrogen) at 37°C in a 0% CO_2_ humidified atmosphere. Cells in the exponential phase of growth were trypsinized with Trypsin-EDTA (Invitrogen), suspended in serum-free medium containing 1% BSA (Sigma-Aldrich) and 200,000 cells per cm^2^ were seeded in 1.9 cm^2^ wells on acidified glass cover-slips coated with PAI-1 14-1b (the stable active form of PAI-1 that shows full inhibitory activity and binds to VN and LRP), PAI-1 R (stable active form with transient inhibitory activity; binds to VN), PAI-1 R76E (stable active form with full inhibitory activity; binds to VN but not to LRP) (kindly provided by Dr. Daniel A Lawrence), or collagen ([Ref. 354236] BD Biosciences) as indicated. In all experiments, unless otherwise stated, PAI-1 was used at the concentration of 20 µg/cm^2^ and collagen at 10 µg/cm^2^. Non-coated acidified glass cover-slips were used for controls. The ROCK inhibitor Y27632 (10 µM; [Ref. Y0503] Sigma-Aldrich), staurosporin (1 µM; [Ref. 9953] Cell Signaling), rabbit anti-uPA (H-140) antibody (15 µg/mL; [Ref. sc-14019] Santa Cruz Biotechnology, Inc.), rabbit anti-uPAR (FL-290) antibody (15 µg/mL; [Ref. sc-10815] Santa Cruz Biotechnology, Inc.), rabbit IgG (15 µg/mL; [Ref. sc-2027] Santa Cruz Biotechnology, Inc.) and soluble PAI-1 14-1b (76 µg/mL, corresponding to 20 µg/cm^2^) were also added to the medium when required.

### Adherence assay

200,000 cells per cm^2^ were seeded in 1.9 cm^2^ wells on acidified glass coverslips coated with PAI-1 14-1b or collagen in Leibovitz L-15 medium supplemented with 1% BSA, 100 U/ml penicillin, 100 µg/mL streptomycin and incubated at 37°C in a 0% CO_2_ humidified atmosphere for 3, 6, 19 and 24 hours. Adherent cells were fixed in 3.65% formaldehyde for 10 min and stained with 0.2% crystal violet at room temperature for 15 min. After solubilization of the crystal violet dye with 1% SDS, the number of adherent cells was calculated by measuring the absorbance at 570 nm with a Sunrise plate reader (Tecan).

### Cell growth assay

200,000 cells per cm^2^ were seeded in 1.9 cm^2^ wells on acidified glass cover-slips coated with PAI-1 14-1b or collagen in Leibovitz L-15 medium supplemented with 1% BSA, 100 U/ml penicillin, 100 µg/mL streptomycin and incubated at 37°C in a 0% CO_2_ humidified atmosphere for 6 hours. Then the medium was replaced by Leibovitz L-15 medium supplemented with 10% FCS, 100 U/ml penicillin and 100 µg/mL streptomycin. After 24, 48 and 72 hours, cells were trypsinized with Trypsin-EDTA (Invitrogen) and counted with a Malassez counting chamber.

### Immunoblotting

Cells were washed in phosphate-buffered saline (PBS) and lysed directly in 50 mM Tris pH 7.5, 150 mM NaCl, 1 mM EDTA, 10% glycerol, 0.5% NP40 supplemented with protease inhibitor cocktail ([Ref. 11836170001] Roche). Denatured proteins were separated by electrophoresis on 12% SDS-polyacrylamide or 4–12% Bis-Tris gels and transferred either to polyvinylidene fluoride or nitrocellulose membranes, then blocked with 5% non-fat dry milk-PBS at room temperature for 1 hour. Membranes were incubated with rabbit anti-MLC-P (Thr 18)-R (dilution 1∶500; [Ref. sc-19848-R] Santa Cruz Biotechnology, Inc.), rabbit anti-PAI-1 (H-135) (dilution 1∶2000; [Ref. sc-8979] Santa Cruz Biotechnology, Inc.) or rabbit anti-cleaved Caspase 3 (dilution 1∶500; [Ref. 9661] Cell Signaling) primary antibodies at 4°C overnight and then with HRP-conjugated goat anti-rabbit secondary antibodies (dilution 1∶2,000; [Ref. sc-2004] Santa Cruz Biotechnology, Inc.) at room temperature for 1 hour. The signal was detected with ECL or ECL^+^ reagents (Amersham Biosciences). Mouse anti-α-Tubulin (dilution 1∶10,000; [Ref. T6199] Sigma-Aldrich) or mouse anti-β-Tubulin (TUB 2.1) antibodies (dilution 1∶2,000; [Ref. sc-58886] Santa Cruz Biotechnology, Inc.) were used to control equal loading and binding was detected with a HRP-conjugated goat anti-mouse secondary antibody (dilution 1∶2,000; [Ref. sc-2005] Santa Cruz Biotechnology, Inc.).

### RhoA activity assay

RhoA activity was assessed as previously described [52]. Cells were washed in PBS and lysed directly in 50 mM Tris pH 7.5, 1% Triton X-100, 0.5% sodium deoxycholate, 500 mM NaCl, 10 mM MgCl_2_, 1 mM DTT, 0.5 mM Vanadate, 1 mM PMSF supplemented with protease inhibitor cocktail ([Ref. P8340] Sigma-Aldrich). Cleared lysates were precipitated with 60 µg of Rhotekin-RBD protein GST beads ([Ref. RT02] Cytoskeleton Inc.) at 4°C for 1 hour. Complexes were washed in buffer containing 50 mM Tris pH 7.5, 1% Triton X-100, 150 mM NaCl, 10 mM MgCl_2_, 1 mM DTT, 1 mM PMSF supplemented with protease inhibitor cocktail, then denatured in Laemmli sample buffer, immunoblotted and incubated with a mouse anti-RhoA antibody (dilution 1∶300; [Ref. sc-418] Santa Cruz Biotechnology, Inc.) followed by a HRP-conjugated sheep anti-mouse secondary antibody (dilution 1∶5,000; [Ref. NA9310] GE Healthcare). An aliquot of the total lysate was run alongside to quantify the total RhoA present in cell lysates in order to determine RhoA activity as the amount of RBD-bound RhoA relative to total RhoA; α-Tubulin expression was used to control for equal loading.

### Immunofluorescence

Cell membranes were stained *in vivo* by incubation with a wheat germ agglutinin Alexa Fluor 488/350 conjugate (dilution 1∶200; [Ref. W11261, W11263] Invitrogen) diluted in HBSS (Invitrogen) at 37°C for 10 min. Cells were then fixed in 3.65% formaldehyde, permeabilized in 0.1% Triton X-100 for 2 min and blocked in PBS 1% BSA at room temperature for 20 min. F-Actin was stained by incubating cells with Phalloidin-TRITC (1 µg/mL; [Ref. P1951] Sigma-Aldrich) at room temperature for 30 min. DNA was stained by incubating cells with 4,6-diamidino-2-phenylindole dihydrochloride (dilution 1∶50,000; [Ref. D1306] Invitrogen) at room temperature for 4 min. The primary antibodies used were mouse anti-β-Tubulin (TUB 2.1) (dilution 1∶200; [Ref. sc-58886]), mouse anti-β1 Integrin (102DF5) (dilution 1∶200; [Ref. sc-73610]), rabbit anti-Ezrin (H-276) (dilution 1∶200; [Ref. sc-20773]), rabbit anti-PAI-1 (H-135) (dilution 1∶200; [Ref. sc-8979]), rabbit anti-uPA (H-140) (dilution 1∶200; [Ref. sc-14019]), mouse anti-uPAR (IID7) (dilution 1∶200; [Ref. sc-32765]) (all from Santa Cruz Biotechnology, Inc.), mouse anti-ROCK1 (46) (dilution 1∶200; [Ref. 611136] BD Biosciences), rabbit anti-PDK1 (Y336) (dilution 1∶200; [Ref. ab32573] Abcam) and rabbit anti-Flotillin1 (dilution 1∶200; [Ref. F1180] Sigma-Aldrich). Cells were incubated at room temperature with the relevant primary antibody for 2 hours and then for 1 hour with TRITC-conjugated (dilution 1∶100; [Ref. sc-3841, sc-3796] Santa Cruz Biotechnology, Inc.) or Alexa Fluor 488-conjugated (dilution 1∶100; [Ref. A11008, A11001] Invitrogen) secondary antibodies. Slides were then mounted in Mowiol solution.

### F-Actin 3D reconstruction

After incubation with Phalloidin-TRITC, the F-Actin cytoskeleton was observed with a 63×/1.40 NA Plan Neofluar objective lens and a laser confocal LSM 410 microscope (Zeiss). Optical sections of cells were recorded with a z-axis of 0.2 µm and individual frames were averaged 8 times with the line mode. Before the 3D reconstruction, the stack of the grey level images (8 bits) was deconvoluted using the module of the Axiovision 3.0.6 software (Zeiss) which allows correcting the distortion due to the optical system of the microscope. The fixed image data were implemented at the appropriate scale with the Amira 2.3 software (TGS Inc.) which uses the threshold segmentation method to retain the details of cell cytoskeleton.

### Microscopy

Phase-contrast images of fixed cells in PBS were taken using a 40×/1.30 objective lens and a microscope equipped with an AxioCam MRm vers.3 camera (Zeiss). Fluorescence images of cells prepared as described before were taken using a 63×/1.30 Plan-Neofluar oil immersion objective lens and a Structured Illumination Microscopy (SIM) (ApoTome Axiovert 200, Zeiss), which is an alternative method for obtaining optical sections, comparable to confocal microscopy. Time-lapse phase-contrast recordings of cells in medium were performed at 37°C and images were taken at intervals of 30 s to 4 min for 5 min to 19 h with a 40×/1.30 objective lens and the acquisition software AxioVision 4.0 (Zeiss).

### Quantification of cell morphology

The different cell morphologies were quantified with the Cell Counter plugin of the ImageJ software (http://rsb.info.nih.gov/ij) using thirty to eighty phase-contrast images taken from three to eight independent experiments (10 images/experiment). Cells were classified in three categories: spindle-shaped, blebbing and round cells.

### Statistical analysis

Student's unpaired *t* tests were performed using QuickCalcs (GraphPad Software; www.graphpad.com/quickcalcs/index.cfm).

### Signal distribution analysis

The signal distribution of a given protein along selected lines in cells was obtained by using the Plot Profile tool of the ImageJ software.

### Colocalization analysis

The colocalization of two proteins was evaluated with the Colocalization tool of the AxioVision software. The percentage of colocalization of two proteins, i.e. the percentage of colocalized pixels relative to the total number of pixels for each detected protein, was obtained by selecting quadrants around cells and fixing a common significant intensity threshold for the two channels (a grey level of 100, with the background set at 25 and the maximal signal at 1000). The mean colocalization percentage of each stained protein and the s.e.m. were calculated by analyzing 10 cells. The colocalization images with the extracted common pixels are shown after the merged images.

### Simulation of PAI-1 deposition

We implemented two *in silico* models to simulate PAI-1 deposition during tumor growth. The cellular-automata model simulates secretion, diffusion and deposition of any diffusible protein and was implemented with the Mathematica software (www.wolfram.com). The agent-based model considers different processes (secretion, deposition and internalization of PAI-1, proteolysis of the matrix and cell proliferation) and was implemented with the NetLogo software (http://ccl.northwestern.edu/netlogo).

## Supporting Information

Figure S1 **Microenvironment influence on cell morphology.** Percentage of spindle-shaped and blebbing SW620 cells seeded in plates coated with different concentrations of PAI-1 14-1b or collagen (from 2 to 40 µg/cm^2^) at different time-points. Data are the mean ± s.e.m. of four independent experiments.
(TIF)

Figure S2 **Cell adherence and cell growth.** (**A**) Number of adherent SW620 cells seeded on plates coated with PAI-1 14-1b or collagen at different time-points (3–24 h) after seeding. Data are the mean ± s.e.m. of four independent experiments; *: *P*<0.05. (**B**) Number of SW620 cells seeded on PAI-1 14-1b or collagen at different time-points (0–72 h) after seeding. Data are the mean ± s.e.m. of four independent experiments. F-Actin (red) and DNA (blue) of SW620 cells seeded on PAI-1 14-1b or collagen for 19 h were immunostained to check for DNA cleavage.
(TIF)

Figure S3 **Expression of components of the Plasminogen Activator system and related signaling molecules varies according to the cell morphology and the microenvironment.** (**A**) PDK1 and ROCK1 expression in blebbing SW620 cells seeded on PAI-1 14-1b or collagen at the 19 h time-point after seeding. On the right panel, quantification of ROCK1/PDK1 colocalization relative to total ROCK1 expression in blebbing SW620 cells seeded on PAI-1 14-1b or collagen. Data are the mean ± s.e.m. of the results of the analysis of ten blebbing cells for each experimental condition. Expression of (**B**) uPA and uPAR, (**C**) PAI-1 and uPAR in blebbing SW620 cells seeded on PAI-1 14-1b at the 19 h time-point after seeding. Analysis of the localization of (**D**) β1 Integrin and PAI-1, (**E**) β1 Integrin and Flotillin1 in SW620 cells seeded on PAI-1 14-1b (30 min time-point). (**F**) Immunostaining of the cell membrane and uPA in SW620 cells seeded on PAI-1 14-1b (30 min time-point). Bar, 5 µm.
(TIF)

Figure S4 **Role of the Plasminogen Activator system in cell migration.** The Plasminogen Activator system is primarily associated with the proteolytic type of cell migration where active uPA catalyzes the cleavage of Plasminogen into Plasmin which in turn facilitates the release of several proteolytic enzymes and the degradation of the extracellular matrix [A] [53], [54]. The principal uPA inhibitor, PAI-1, inhibits the proteolytic activity of uPA [B] [55]. When PAI-1 is bound to Vitronectin (VN), a major matrix component, and uPA to its surface receptor uPAR, the [uPA∶PAI-1] complex forms a molecular link between cell and matrix that can allow the transmission of an intracellular mechanical signal [C] [32] and results in the internalization of the whole complex by the α2-Macroglobulin Receptor/Low-density Lipoprotein Receptor (α2-MR/LRP) [D] [35], [56]. Then the [uPA∶PAI-1] complex is degraded by lysosomes [E] [55], [57] and uPAR and LRP are recycled [F] [35]. uPAR also promotes cell adhesion through its interaction with VN in the extracellular matrix and can transmit an intracellular biochemical signal via Integrins [G] [27]. However, active PAI-1, independently of its role as a protease inhibitor, inhibits uPAR adhesion by blocking uPAR binding to VN [H] [16], [18]. Active PAI-1 also inhibits Integrin- and VN-mediated cell migration by blocking binding of αvβ3 Integrin to VN [I] [19], [54].
(TIF)

Figure S5 **Agent-based simulation of matrix-bound PAI-1 accumulation at the tumor periphery.** In this simulation several physiological processes, such as PAI-1 secretion, deposition and internalization, proteolysis of the matrix and cell proliferation are taken into account (see also Video S5).
(TIF)

Video S1 **Motility of blebbing SW620 cells seeded on immobilized, active PAI-1.** Time-lapse recording of blebbing SW620 cells 3 h after seeding on PAI-1 14-1b. Images were captured every 2 min. Bar, 10 µm.
(MOV)

Video S2 **PAI-1 expression in SW620 cells seeded on non-coated plates.** The z-stack video shows details of the immunostaining of the cell membrane (red) and of PAI-1 expression (green) from the slide to the upper part of a SW620 cell seeded on non-coated plate for 30 min. Bar, 5 µm.
(MOV)

Video S3 **Flotillin1 and PAI-1 expression in SW620 cells seeded on non-coated plates.** The z-stack video shows details of Flotillin1 (red) and PAI-1 (green) from the slide to the upper part of a SW620 cell seeded on non-coated plate for 30 min. Bar, 5 µm.
(MOV)

Video S4 **PDK1 and ROCK1 expression in SW620 cells seeded on immobilized, active PAI-1.** The z-stack video shows details of PDK1 (red) and ROCK1 (green) from the slide to the upper part of a SW620 cell seeded on PAI-1 14-1b for 30 min. Bar, 5 µm.
(MOV)

Video S5 **Agent-based simulation of matrix-bound PAI-1 accumulation at the tumor periphery.** Several physiological processes are considered such as secretion, deposition and internalization of PAI-1, proteolysis of the matrix and cell proliferation. See color legend in Figure S5.
(MOV)

Video S6 **Migration of a blebbing SW620 cell on immobilized, active PAI-1.** Time-lapse recording of SW620 cells 17 h after seeding on PAI-1 14-1b. The migration speed was evaluated at 0.4 µm/min. Images were captured every 2 min. Bar, 10 µm.
(MOV)

Video S7 **Reversibility of the blebbing and spindle-shaped morphologies in SW620 cells seeded on immobilized, active PAI-1.** Time-lapse recording of SW620 cells 7 h after seeding on PAI-1 14-1b. Images were captured every 2 min. Bar, 10 µm.
(MOV)

Video S8 **Reversibility of the blebbing morphology in SW620 cells seeded on immobilized, active PAI-1.** Time-lapse recording of SW620 cells 16 h after seeding on PAI-1 14-1b. Images were captured every 2 min. Bar, 10 µm.
(MOV)
