# Supplementary figures and images for: Matrix-Bound PAI-1 Supports Cell Blebbing via RhoA/ROCK1 Signaling
Source: PLoS One. 2012 Feb 21;7(2):e32204. doi: 10.1371/journal.pone.0032204 (PMC3283740; doi:10.1371/journal.pone.0032204)

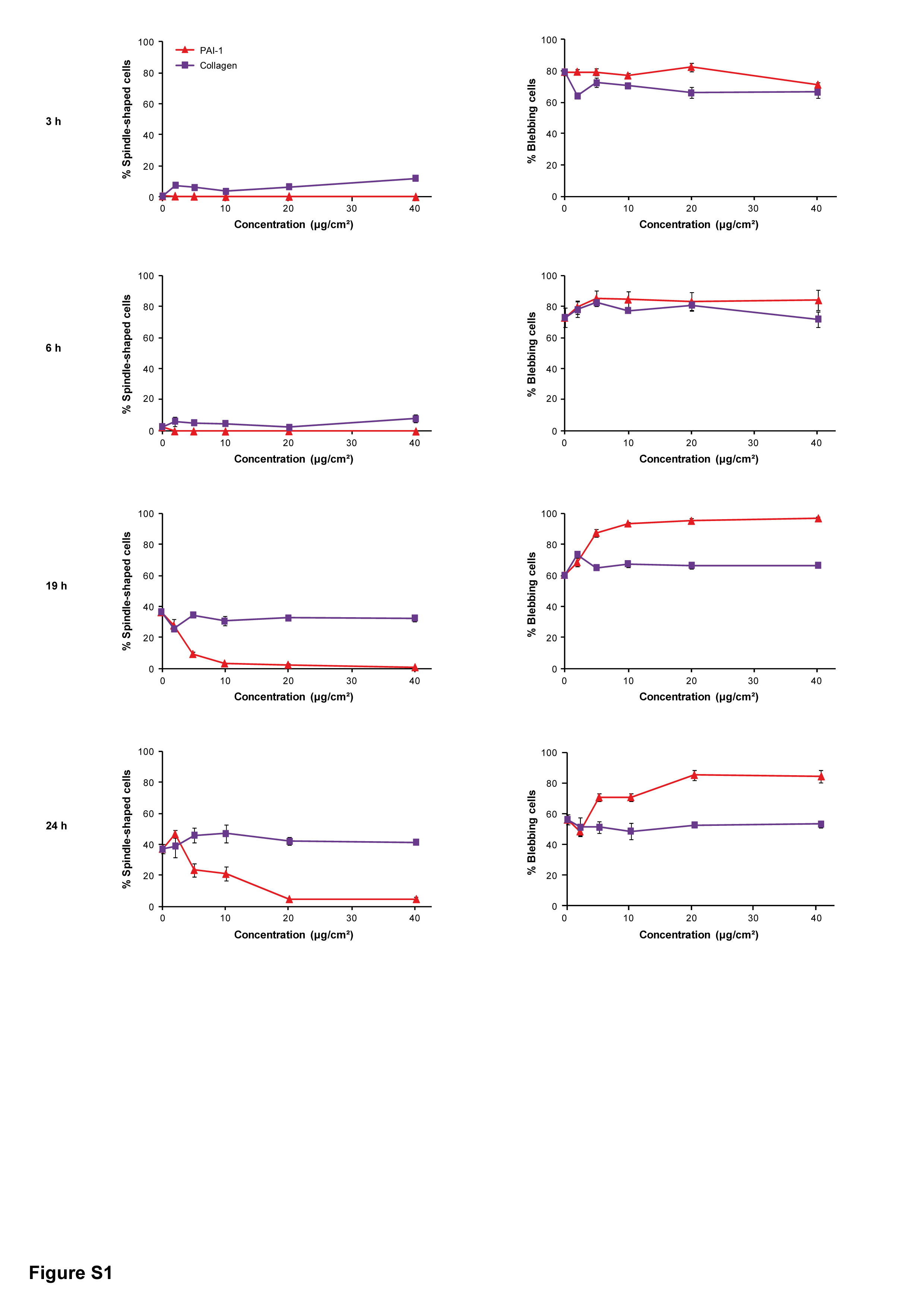

Supplement: Figure S1 — Microenvironment influence on cell morphology. Percentage of spindle-shaped and blebbing SW620 cells seeded in plates coated with different concentrations of PAI-1 14-1b or collagen (from 2 to 40 µg/cm2) at different time-points. Data are the mean ± s.e.m. of four independent experiments. (TIF) [file pone.0032204.s001.tif]

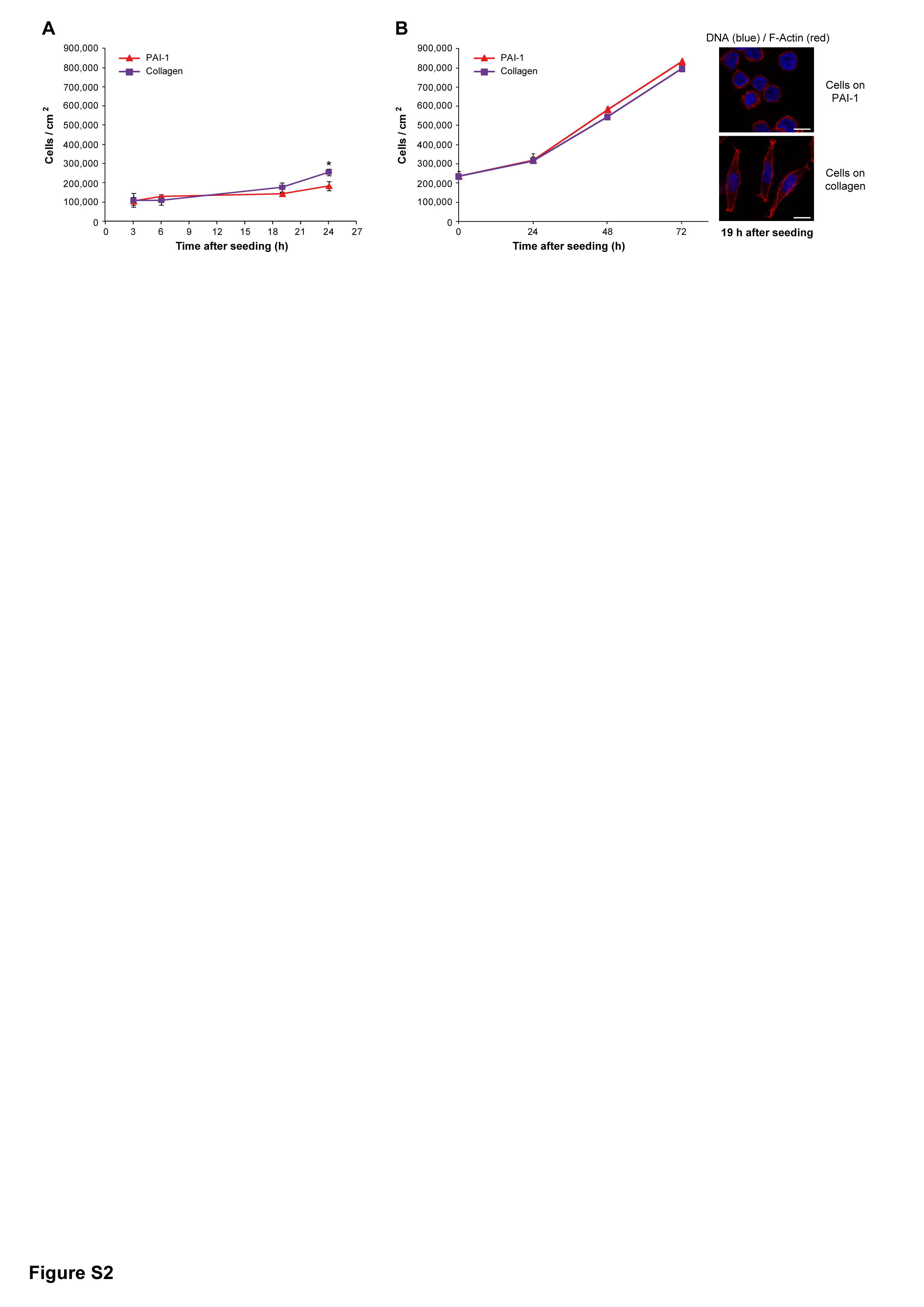

Supplement: Figure S2 — Cell adherence and cell growth. (A) Number of adherent SW620 cells seeded on plates coated with PAI-1 14-1b or collagen at different time-points (3–24 h) after seeding. Data are the mean ± s.e.m. of four independent experiments; *: P<0.05. (B) Number of SW620 cells seeded on PAI-1 14-1b or collagen at different time-points (0–72 h) after seeding. Data are the mean ± s.e.m. of four independent experiments. F-Actin (red) and DNA (blue) of SW620 cells seeded on PAI-1 14-1b or collagen for 19 h were immunostained to check for DNA cleavage. (TIF) [file pone.0032204.s002.tif]

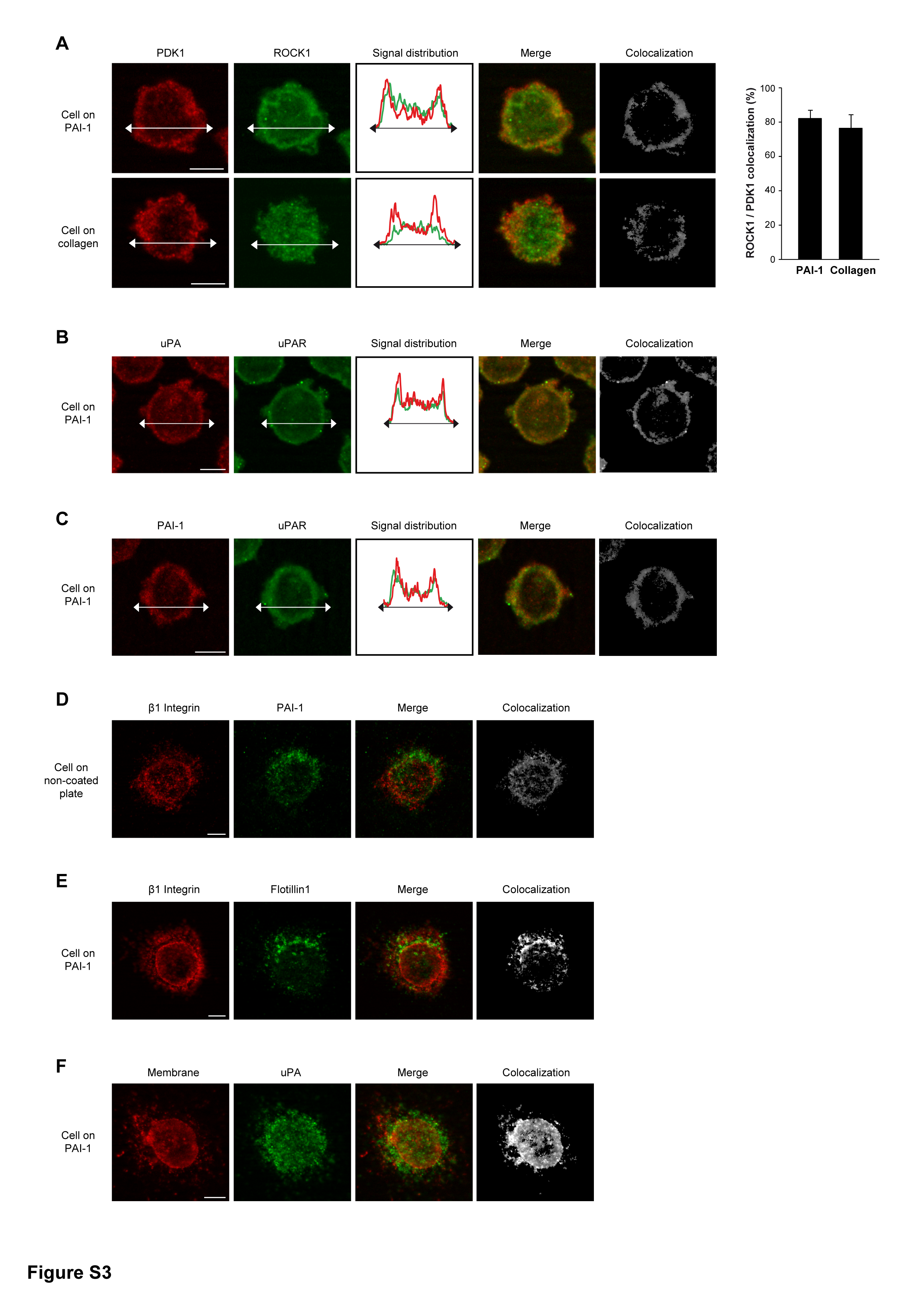

Supplement: Figure S3 — Expression of components of the Plasminogen Activator system and related signaling molecules varies according to the cell morphology and the microenvironment. (A) PDK1 and ROCK1 expression in blebbing SW620 cells seeded on PAI-1 14-1b or collagen at the 19 h time-point after seeding. On the right panel, quantification of ROCK1/PDK1 colocalization relative to total ROCK1 expression in blebbing SW620 cells seeded on PAI-1 14-1b or collagen. Data are the mean ± s.e.m. of the results of the analysis of ten blebbing cells for each experimental condition. Expression of (B) uPA and uPAR, (C) PAI-1 and uPAR in blebbing SW620 cells seeded on PAI-1 14-1b at the 19 h time-point after seeding. Analysis of the localization of (D) β1 Integrin and PAI-1, (E) β1 Integrin and Flotillin1 in SW620 cells seeded on PAI-1 14-1b (30 min time-point). (F) Immunostaining of the cell membrane and uPA in SW620 cells seeded on PAI-1 14-1b (30 min time-point). Bar, 5 µm. (TIF) [file pone.0032204.s003.tif]

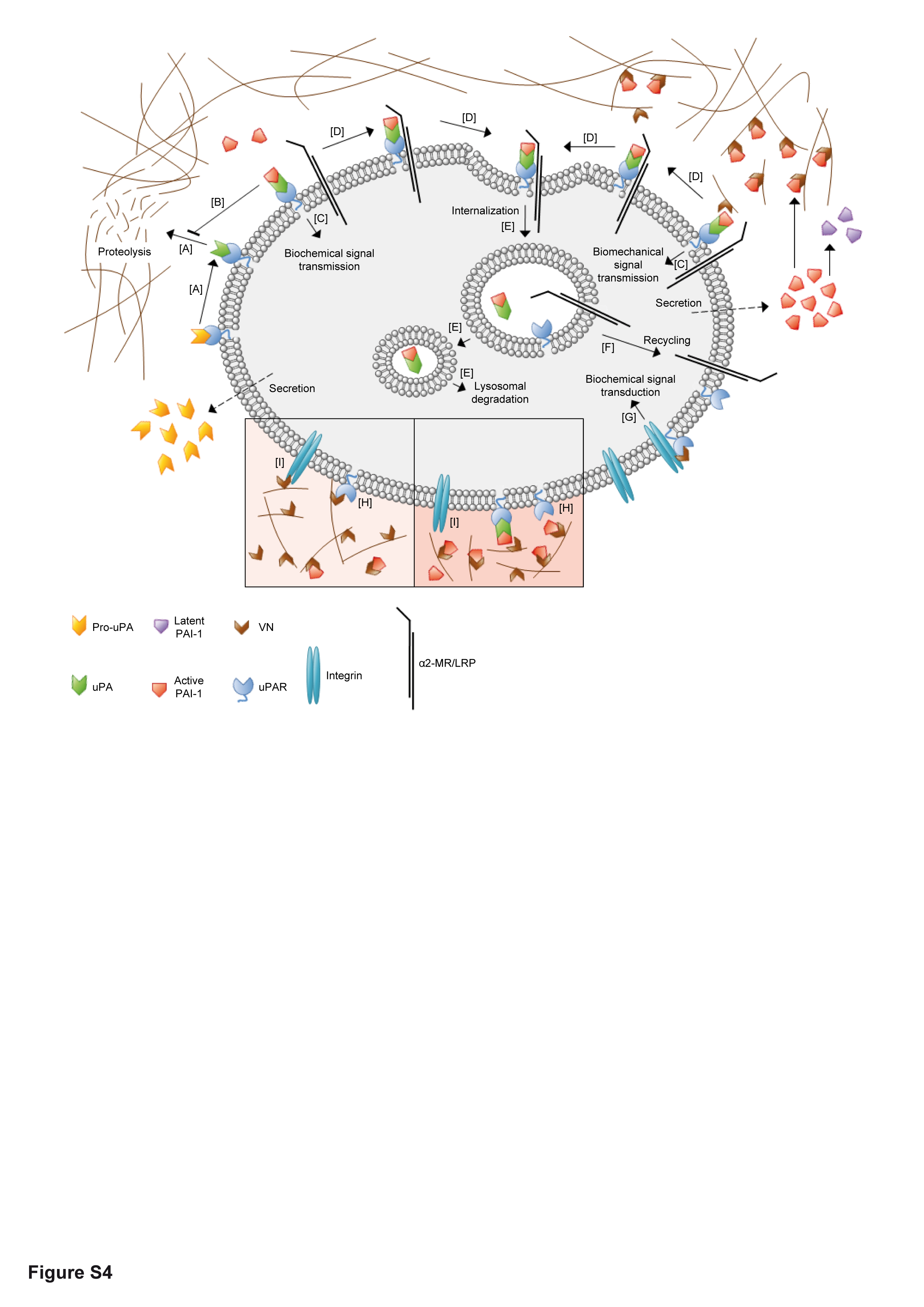

Supplement: Figure S4 — Role of the Plasminogen Activator system in cell migration. The Plasminogen Activator system is primarily associated with the proteolytic type of cell migration where active uPA catalyzes the cleavage of Plasminogen into Plasmin which in turn facilitates the release of several proteolytic enzymes and the degradation of the extracellular matrix [A] [53], [54]. The principal uPA inhibitor, PAI-1, inhibits the proteolytic activity of uPA [B] [55]. When PAI-1 is bound to Vitronectin (VN), a major matrix component, and uPA to its surface receptor uPAR, the [uPA∶PAI-1] complex forms a molecular link between cell and matrix that can allow the transmission of an intracellular mechanical signal [C] [32] and results in the internalization of the whole complex by the α2-Macroglobulin Receptor/Low-density Lipoprotein Receptor (α2-MR/LRP) [D] [35], [56]. Then the [uPA∶PAI-1] complex is degraded by lysosomes [E] [55], [57] and uPAR and LRP are recycled [F] [35]. uPAR also promotes cell adhesion through its interaction with VN in the extracellular matrix and can transmit an intracellular biochemical signal via Integrins [G] [27]. However, active PAI-1, independently of its role as a protease inhibitor, inhibits uPAR adhesion by blocking uPAR binding to VN [H] [16], [18]. Active PAI-1 also inhibits Integrin- and VN-mediated cell migration by blocking binding of αvβ3 Integrin to VN [I] [19], [54]. (TIF) [file pone.0032204.s004.tif]

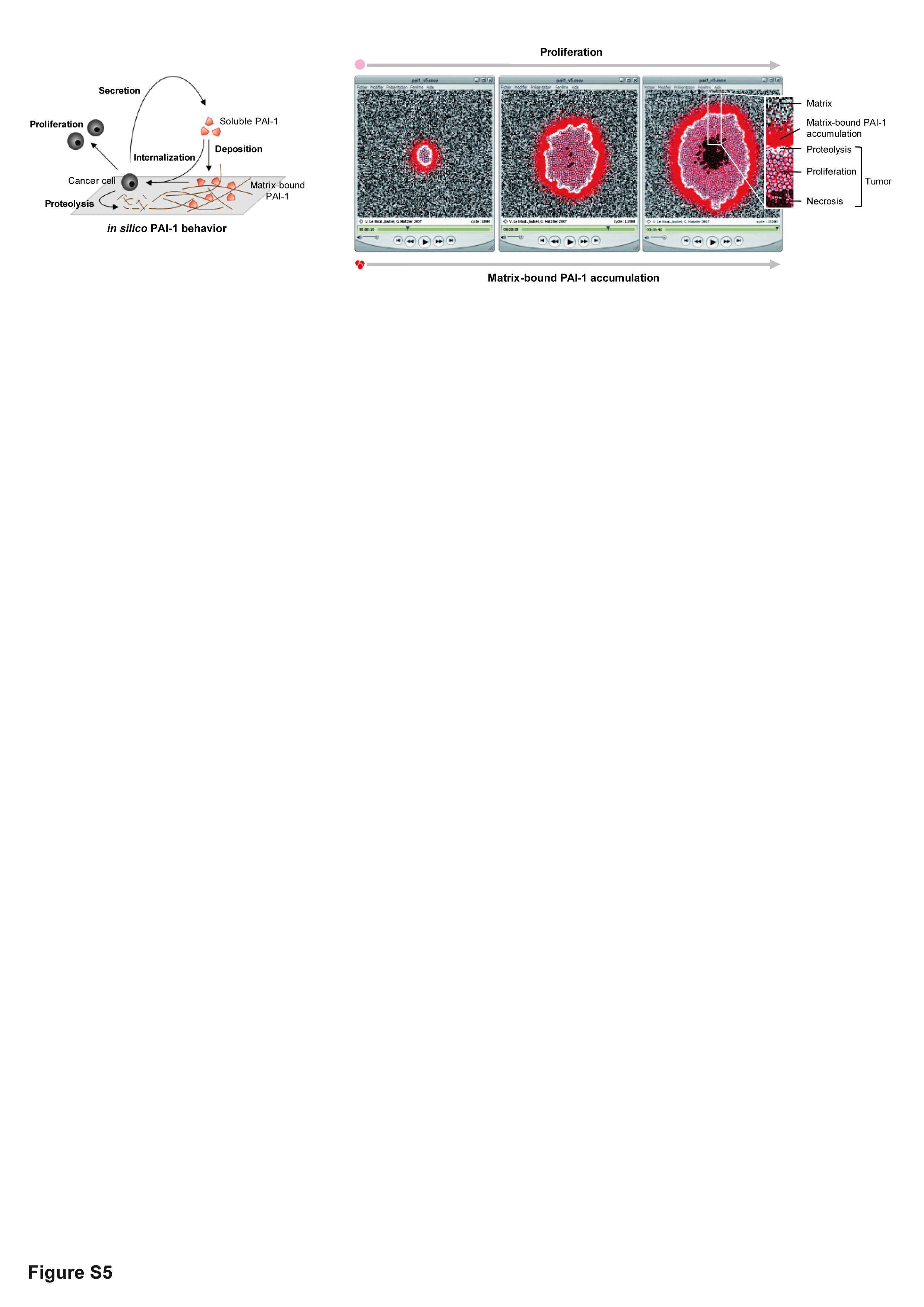

Supplement: Figure S5 — Agent-based simulation of matrix-bound PAI-1 accumulation at the tumor periphery. In this simulation several physiological processes, such as PAI-1 secretion, deposition and internalization, proteolysis of the matrix and cell proliferation are taken into account (see also Video S5). (TIF) [file pone.0032204.s005.tif]
